# Co-evolution of private and public hospitals: spatiotemporal disparities, geospatial interactions, and social determinants over 19 years in Sichuan, China

**DOI:** 10.3389/fpubh.2025.1644657

**Published:** 2025-12-08

**Authors:** Xiao Liu, Xiuli Wang, Yaqian He, Xianteng Tang, Jinghua Wang, Xiange An, Lingfeng Liao, Xiang Yan, Yumeng Zhang, Chao Song

**Affiliations:** 1 HEOA–West China Health & Medical Geography Group, West China School of Public Health and West China Fourth Hospital, Sichuan University, Chengdu, China; 2 Institute for Healthy Cities and West China Research Center for Rural Health Development, Sichuan University, Chengdu, China; 3 Health Promotion and Food Nutrition & Safety Key Laboratory of Sichuan Province, Chengdu, China; 4 Department of Geosciences, University of Arkansas, Fayetteville, AR, United States; 5 State Key Laboratory of Oil and Gas Reservoir Geology and Exploitation, School of Geoscience and Technology, Southwest Petroleum University, Chengdu, China; 6 College of Architecture and Environment, Institute of Urbanization Strategy and Architecture Research, Sichuan University, Chengdu, China

**Keywords:** private hospital, public hospital, spatiotemporal disparity, geospatial interaction, social determinant, three-level healthcare system, China

## Abstract

**Background:**

The global rise of private hospitals is crucial for achieving universal health coverage, yet the development of public and private hospitals remains uncoordinated. This study explores the co-evolution of private and public hospitals, focusing on their spatiotemporal disparities, geospatial interactions, and the social determinants under policy guidance.

**Methods:**

We used Sichuan province, China, as a case study and collected hospital-level annual report data from 2002 to 2020. Spatiotemporal analyses examined the co-evolution of public and private hospitals across different hierarchical levels. The Gini coefficient assessed the spatial equity of hospital bed resources, while spatial accessibility was measured using the provider-to-population ratio at district and county levels. Trend analysis quantified changes in accessibility over time. Fixed-effects models identified social determinants influencing hospital resource allocation.

**Results:**

Between 2002 and 2020, the proportion of districts/counties in Sichuan with more than 4.8 hospital beds per 1,000 population increased significantly, from 5.46% to 43.72%. The equity of medical bed resources also improved across the province. The proportion of districts/counties with more than 3.3 public hospital beds per 1,000 population rose from 12.57% to 50.27%, and the share of districts/counties where private hospitals made up 25% or more of total beds grew from 2.19% to 53.01%. Geospatial interaction maps revealed regional disparities: complementarity in advantaged areas, persistent deficits in remote regions, and geographical compression of public hospitals in urban centers. Our analysis further showed that private hospital accessibility positively correlates with population density, per capita GDP, and government health expenditure, while public hospital accessibility is positively linked to per capita GDP, urbanization, and health expenditure. However, public primary hospital accessibility negatively correlates with per capita GDP.

**Discussion:**

While private hospitals have rapidly expanded bed capacity, policy biases and market incentives have caused a structural imbalance, with a shortage of high-end services and an excess of low-end resources. In contrast, public hospitals have upgraded hierarchically, concentrating high-quality resources in urban areas. However, basic medical supply remains insufficient in remote regions, exacerbating disparities in healthcare accessibility and quality, and hindering the achievement of universal health coverage.

## 1 Introduction

In recent decades, the global healthcare landscape has undergone a significant transformation, marked by the rapid expansion of the private hospital sector (1). By 2020, the number of private hospital beds had surpassed those of public hospitals in numerous countries, such as Japan and the United Kingdom (2, 3). This development has positioned private hospitals as an indispensable component of modern healthcare systems. In many low- and middle-income countries (LMICs), constrained public health budgets often result in insufficient healthcare resources. The development of private hospitals, driven by both governmental policies and market forces, has not only enhanced the overall provision of services but also helped mitigate health equity challenges by improving access to healthcare (4). Consequently, private hospitals have become increasingly important in achieving universal health coverage (UHC) (5–7).

Despite this growth, there is currently no international consensus on the optimal structure of healthcare markets. In Latin American countries, public hospitals dominate the healthcare system (8). However, following the introduction of private hospitals in Chile, the healthcare system experienced decreased efficiency and increased medical costs (9). In contrast, countries such as India and Pakistan rely heavily on the private sector for healthcare services due to low public health expenditure, which has led to high out-of-pocket payments and severe health poverty (10, 11). While existing literature often frames the government guidance and market competition as mutually exclusive (12), recent theoretical advancements suggest that their interaction is highly context-dependent (13). According to World Bank assessments of LMICs, a purely public healthcare delivery system is prone to “government failure,” while a fully market-oriented model may exacerbate “market failure.” The expansion of private hospitals in England, for example, enhanced public sector services without improving quality, demonstrating a symbiotic rather than adversarial relationship between the two sectors (14, 15).

Previous studies have confirmed that the unregulated expansion of private hospitals tends to exacerbate spatial inequalities in healthcare resources (16). The persistent dilemma remains: the public sector alone lacks the fiscal and human resources to achieve UHC. In response, global policy actors including the International Finance Corporation (IFC), the World Health Organization (WHO), and the United States Agency for International Development (USAID), as well as industry associations, advocate for cross-sectoral integration. This approach seeks to engage the private healthcare sector as a constructive partner in enhancing the accessibility and equity of medical services (17).

By fostering a healthcare system in which public and private hospitals coexist, a hybrid complementary-competitive mechanism formed through cooperative or competitive market entry can be established. This model integrates innovation incentives from market entities while maintaining public accountability, thereby enhancing both the accessibility and quality of healthcare services (18, 19). Recent years have seen increasing focus on the equitable distribution of geographically constrained resources to meet healthcare needs in medical service planning and resource allocation (20, 21). Although the Managed Competition model is designed to achieve equity by explicitly combining public regulation and private efficiency incentives, its actual spatial effects, especially in low-density areas, remain a subject of debate (22). By examining the co-evolution of public and private institutions within the healthcare system, and contrasting the spatial accessibility, equity, and quality of care in different regions, particularly underserved and poor areas, can provide empirical evidence for these ongoing debates.

China’s healthcare system allows citizens to access both public and private hospitals and has undergone distinct developmental phases in balancing the roles of government and the market, accumulating substantial practical experience. From 1978 to 1997, the system was characterized by market-oriented reform with weak government oversight. The period from 1997 to 2009 saw the emergence of a mixed public–private system with a diluted welfare function in public hospitals (23). Since 2009, the system has gradually transitioned toward a public-dominated, private-supplemented model (24). Public hospitals in China are government-run, non-profit institutions funded by state subsidies and service fees. They deliver essential healthcare, drive innovation, and train professionals, but face challenges such as regional disparities in resources, limited specialized care, an inherent inability to achieve universal health coverage independently, and a rising demand for personalized services (5). Private hospitals, which include both for-profit and non-profit entities, operate under varying ownership structures. For-profit hospitals are driven by market principles and flexible pricing (25), while non-profit hospitals focus on public welfare goals. Both public and private hospitals follow similar health insurance payment and reimbursement procedures in basic medical services, and referrals between them are increasingly common within the tiered care system (26). In this context, the inclusion of private hospitals helps to supplement specialized medical resources, reduce healthcare costs, and improve service efficiency, working alongside the public system to enhance accessibility and equity (27).

The year 2020 marked the conclusion of China’s 13th Five-Year Plan and the strategic initiation of the 14th, coinciding with a pivotal transition in the development of private hospitals from quantity-driven expansion to quality-oriented growth. This policy shift offers an important opportunity to systematically evaluate the development trajectory of private hospitals within the overall health system and their evolving relationship with public institutions. LMICs commonly face structural challenges such as scarce public health resources, weak infrastructure, uneven service quality, and inadequate regulatory capacity. Within this context, effectively leveraging private sector resources through sound policy and institutional design has become a crucial issue for improving the accessibility, quality, and equity of healthcare. This study focuses on Sichuan province, a representative case in China’s private hospital reform, offering valuable insights into both the achievements and challenges of phased policy implementation. Through an in-depth analysis of this typical case, the research aims to address gap in understanding the developmental pathway of private hospitals and their collaborative evolution with public hospitals within the broader health system. These findings offer practical implications for optimizing healthcare equity and promoting institutional reforms in China, a country with one-fifth of the world’s population.

Drawing on hospital-level longitudinal data from 2002 to 2020, the study employs statistical analysis and geographic information systems (GIS) to investigate both structural and spatial dynamics across quantitative indicators and qualitative hospital tiers. Through a 19-year longitudinal analysis traces evolving patterns in hospital system architecture, spatial distribution, spatial accessibility and equity under the tiered healthcare delivery framework. Specifically, we examine the co-evolutionary trajectories of public and private hospitals under policy-driven transformation, focusing on the coordination of resource growth between the two sectors. By integrating spatial resource allocation patterns, tier-specific service disparities, and the social determinants of resource distribution, this study provides evidence-based insights to inform health policy design.

## 2 Policies, materials and methods

### 2.1 Healthcare policy reforms of hospitals in China

Since 1978, the development of China’s private hospitals has unfolded over four decades (28), closely intertwined with the evolution of its broader healthcare system. Following the reform and opening-up policy, China began transitioning toward a hybrid public-private healthcare system (29). However, in the early years, the expansion of private hospitals was hindered by limited government support and the over-commercialization of public hospitals (30). Renewed momentum for private hospital reform emerged following the 2002 SARS outbreak, as it triggered widespread recognition of systemic weaknesses in China’s healthcare system (31).

In 2009, the launch of China’s new healthcare reform, outlined in the Opinions on Deepening the Reform of the Medical and Healthcare System, formally emphasized the coordinated development of public and private healthcare institutions (32). The 2010 Notice on Encouraging and Guiding Social Capital in Healthcare eased market entry restrictions and expanded access to public insurance reimbursement, especially encouraging nonprofit private hospitals in underserved regions to support basic healthcare delivery (33). Pilot reforms in 2011 focused on enhancing policy frameworks, ensuring equitable development opportunities for private hospitals, and optimizing their operational conditions (34). In 2014, Sichuan province issued the Several Opinions on Accelerating the Development of Socially Run Healthcare, setting a target for private hospitals to reach 25% of total beds and service volume by 2017. National policies in 2015 and the 13th Five-Year Plan for Health and Wellness in 2016 further supported the private sector through land use and tax incentives, and mandated local governments to reserve planning space for at least 1.5 private hospital beds per 1,000 population (33).

In parallel, public hospital reform advanced as part of broader health system restructuring. Beginning in 2009, China initiated efforts to cap the size of individual public hospitals and reduce their proportion within the system. In 2011, public hospital pilot reforms began to optimize the distribution of public hospitals and to ensure that the government effectively operates at least one county-level hospital in every county. The 2013 Several Opinions on Accelerating the Development of Non-Public Healthcare providers emphasized overall control, structural adjustment, and moderate scale, and called for strict control over the quantity of public hospitals to reserve sufficient development space for private hospitals. The National Health Service System Planning Outline (2015–2020) set a national target of 4.8 beds per 1,000 population by 2020, with 3.3 allocated to public hospitals and a minimum of 1.5 to private hospitals (35). In 2019, policy directives promoted functional differentiation and collaboration between public and private hospitals, with tertiary public hospitals playing a leadership role, and performance evaluations were introduced for tertiary public hospitals, alongside measures to curb excessive hospital bed expansion.

**Figure 1:**
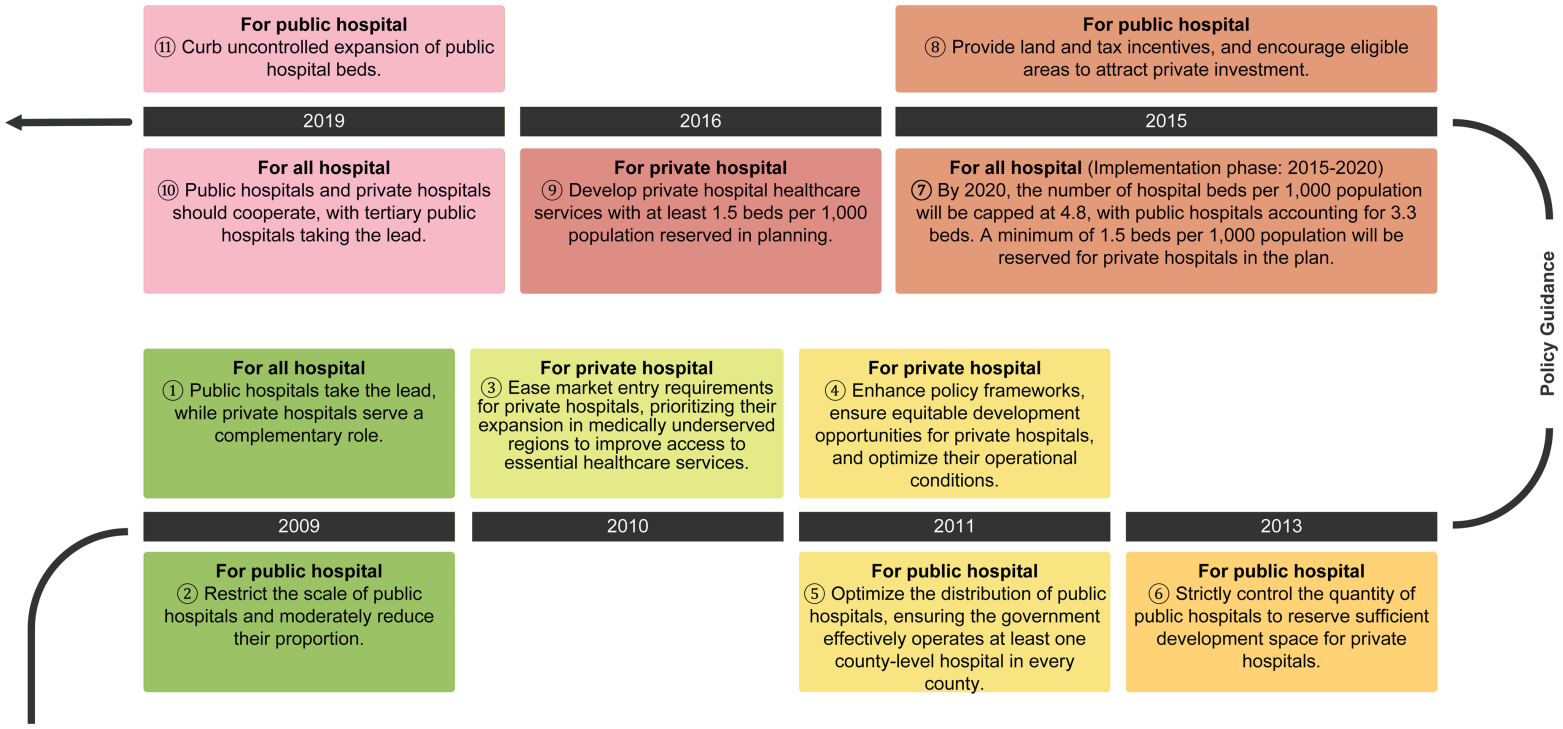
Flowchart of health-system reforms shaping public and private hospitals in China.

Collectively, these reforms reflect a gradual policy shift from unregulated growth to structured coordination between public and private sectors (Figure 1), aiming to improve the efficiency, quality, and equity of healthcare provision across China.

### 2.2 Study area and data

Since 2015, Sichuan province has ranked first in China in the number of private hospitals, offering a substantial pool of institutions that provides a rich empirical foundation for research. As China’s fifth-largest province, Sichuan displays representative characteristics in healthcare resource allocation, service quality, and policy implementation, making it an ideal case study for examining the evolving role of private hospitals within the national healthcare system. Since 1978, the province has closely aligned with central government directives, effectively reflecting national policy trends and implementation outcomes while minimizing interference from local policy deviations, thereby offering an objective basis for policy evaluation and refinement.

**Figure 2:**
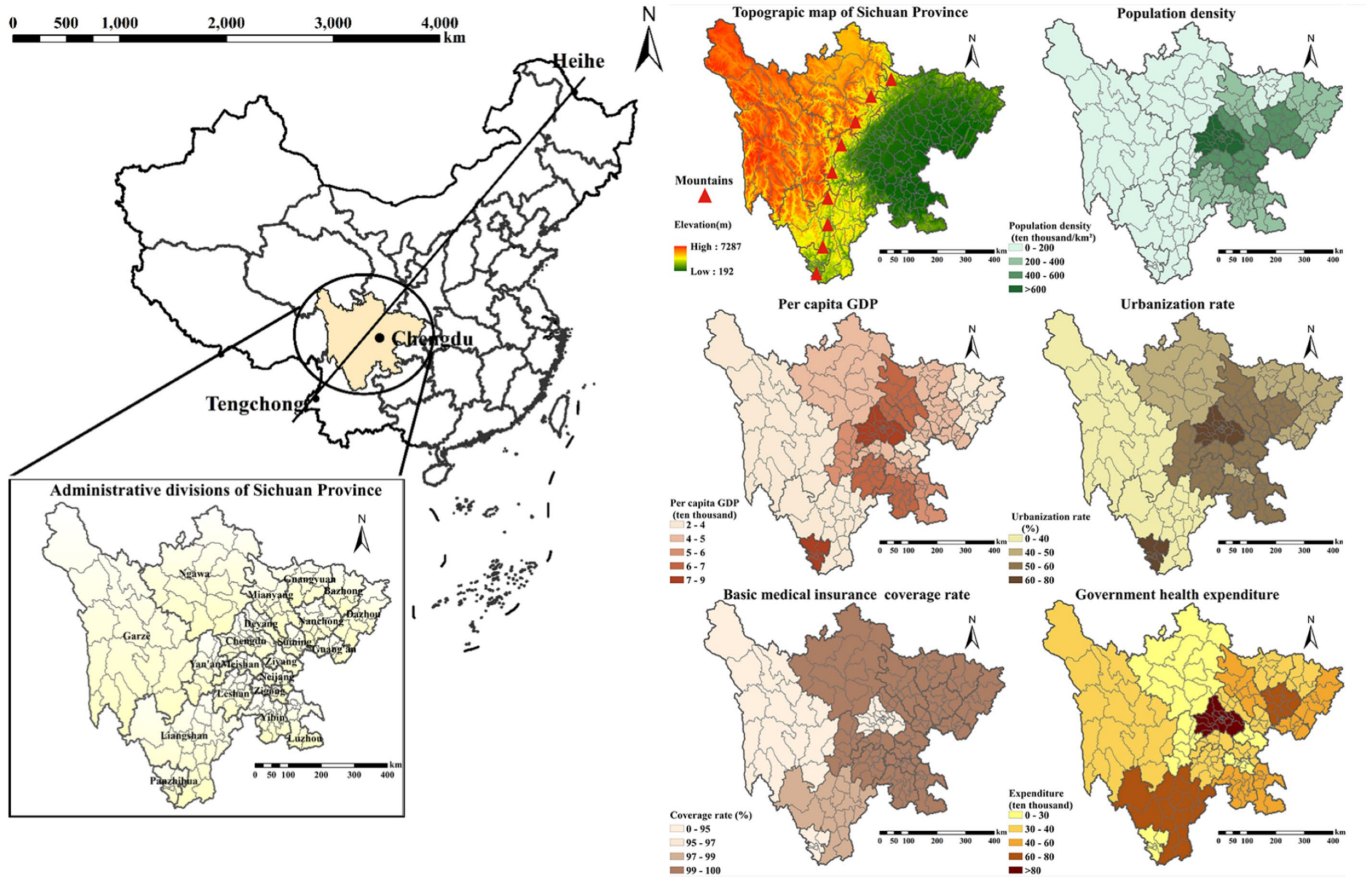
County-level geospatial patterns of the study area and key socioeconomic variables in Sichuan Province, China: population density, per-capita GDP, urbanization rate, basic medical-insurance coverage, and government health expenditure.

Sichuan also exhibits pronounced internal socioeconomic disparities, encompassing both economically developed urban centers such as Chengdu and underdeveloped rural regions. This diversity enables comparative analysis of private hospital development strategies across varying economic contexts. Geographically, the province spans China’s first and second topographic steps and is bisected by the Hengduan Mountains (Figure 2), creating significant regional heterogeneity that supports investigations into geographic influences on private healthcare development. Demographically, Sichuan’s population of 83.71 million in 2020 is widely dispersed, presenting both challenges and opportunities for private hospitals in terms of market penetration and service coverage across populations with varying characteristics.

Annual hospital report data from 2002 to 2020 were obtained from the Sichuan Provincial Health Commission, serving as supply-side data. Each hospital was assigned a unique identifier and geocoded as a point feature in a GIS database using coordinates derived via the Amap API based on registered addresses. Hospitals were categorized into six types based on ownership (public vs. private) and official service level (primary, secondary, tertiary), with the number of hospital beds serving as a proxy for service capacity (36). Ownership classification was based on registration status (37). Public hospitals are typically government-managed institutions that provide essential health services, serve as hubs for medical innovation, and train healthcare professionals. Private hospitals encompass wholly private entities, joint ventures, Sino-foreign partnerships, and other non-governmental ownership types.

Hospital levels were obtained from official classifications in the annual reports, assessed using strict and standardized procedures by government authorities. For ungraded hospitals, classification was inferred based on bed count, following the Hospital Grading Management Standards: hospitals with 20–99 beds were classified as primary, 100–499 as secondary, and ≥ 500 as tertiary. Over the study period, 4,856 hospitals were identified, including 2,503 private hospitals (2,117 primary, 376 secondary, 10 tertiary) and 2,353 public hospitals (1,260 primary, 887 secondary, 206 tertiary). However, only 817 hospitals remained in operation throughout the entire study period, of which fewer than 10% (78 hospitals) were private.

Administrative boundary data for Sichuan were obtained from the National Basic Geographic Information Center. Socioeconomic data covering the 21 prefecture-level administrative divisions from 2002 to 2020 were sourced from the Sichuan Statistical Yearbooks, including population density (PD), per capita gross domestic product (GDP), urbanization rate (UR), basic medical insurance coverage rate (BMICR), and government health expenditure (GHE). And the permanent population data of 183 county-level administrative divisions were obtained from Sichuan Provincial Health Information Center. Specifically, China’s current administrative divisions are divided into five levels, prefecture-level administrative divisions belong to the third level, while county-level administrative divisions serve as the fourth level of administrative units, and each prefecture-level division governing a number of districts/counties as lower-level administrative units. In this study, all spatial accessibility of hospital beds’ analyses were conducted at the prefecture level; concurrently, comparisons of inequality in the distribution of medical resources were also performed at the county level where applicable.

### 2.3 Methods

In this study, descriptive statistics were employed to examine trends in the number, proportion, and spatiotemporal distribution of public and private hospitals at different service levels (primary, secondary, and tertiary) in Sichuan province from 2002 to 2020. Additionally, temporal changes in hospital bed capacity were analyzed to reflect evolving service capacity. The spatiotemporal results were visualized using a series of graphs and thematic maps to clearly illustrate structural shifts in healthcare provision and the influence of policy changes on institutional composition within the healthcare system.

The Gini coefficient was adopted to evaluate the equity of spatial access to different types of hospitals at the prefecture level from 2002 to 2020 using the following formula:

$$\mathrm{Gin}i_{\mathrm{ip}}=1-\sum_{j-1}^{\mathrm{ni}}(X_{j,i,p}-X_{j-1,i,p})(Y_{j,i,p}+Y_{j-1,i,p})$$ (1)

where $\mathrm{Gin}i_{\mathrm{ip}}$ was the Gini coefficient of prefecture *i* (*i* [1,21]) in the year ( p∈ [1,19]), *ni* is the number of districts/counties in prefecture *i*, *j* is the ranking index of the districts/counties in prefecture *q* sorted in ascending order by per capita bed resource availability, $X_{j,i,p}$ is the cumulative percentage of the population in each prefecture in year *p*, and $Y_{j,i,p}$ is the cumulative percentage of bed numbers in each prefecture, where $X_{0}=0$ and $Y_{0}=0$ . Based on Equation 1, we computed the Gini coefficient in R 4.2.1 software environment. It ranges from 0 to 1, with lower values indicating greater equity. Typically, a value below 0.2 suggests equitable resource allocation, 0.2–0.3 indicates relatively equitable, 0.3–0.4 represents somewhat equitable, 0.4–0.5 suggests substantial disparities, and a value greater than 0.5 indicates significant inequalities (38).

The provider-to-population ratio (PPR) is a widely used indicator for assessing the spatial accessibility of healthcare services (39). Without considering cross-border healthcare-seeking behavior, it quantifies healthcare resource availability by measuring the number of healthcare providers or service units (e.g., beds) per population within a given geographic unit (40). In this study, we calculated the number of hospital beds per 1,000 population at the district and county levels across Sichuan province to assess the availability of healthcare resources. Spatial analysis was conducted using ArcGIS to map the spatial accessibility of hospital beds for each of the six hospital categories (public/private × primary/secondary/tertiary) over the 19-year period. The spatial patterns revealed areas of relative abundance or scarcity, helping to identify disparities and inform future healthcare resource planning (41).

The slope trend analysis was applied to evaluate the temporal dynamics and spatial disparities of bed accessibility across prefectures (36, 42). This method enables the identification of long-term changes in healthcare service availability over time and across space. Specifically, the *Slope* value of the provider-to-population ratio over the study period was calculated for each prefecture using the following equation:

$$\text{Slop}e_{\mathrm{ip}}=\frac{m\sum_{p=1}^{m}(p\times A_{\mathrm{ip}})-\sum_{p=1}^{m}p\times \sum_{p=1}^{m}A_{\mathrm{ip}}}{m\sum_{p=1}^{m}p^{2}-{\left(\sum_{p=1}^{m}p\right)}^{2}}$$ (2)

where $\text{Slop}e_{\mathrm{ip}}$ represents the changing trend of spatial access to hospital beds (including general and specialized categories across the six types of hospitals) for prefecture in year ( p∈ [1,19]), $A_{\mathrm{ip}}$ denotes the spatial accessibility of prefecture *i* in year *p*, and *m* corresponds to the duration of the study period (m=19) . Using Equation 2, we derived the Slope value. A positive *Slope* value indicates an upward trend, whereas a negative *Slope* value suggests a downward trend.

To explore the underlying social determinants associated with changes in hospital bed accessibility, a panel data fixed-effects model was employed (43). This model accounts for unobserved time-invariant heterogeneity across prefectures and isolates the influence of time-varying predictors, thereby being able to capture the spatiotemporal heterogeneous impact of potential social factors on hospital beds, that is of crucial importance (44). The dependent variable was the provider-to-population ratio (PPR), and the explanatory variables included prefecture-level indicators from 2002 to 2020: population density (PD), per capita gross domestic product (GDP), urbanization rate (UR), basic medical insurance coverage rate (BMICR) and government health expenditure (GHE). All explanatory variables were confirmed by VIF diagnostics to lie below the threshold (VIF < 10), indicating to be free of multicollinearity (45, 46). The model was estimated using the R 4.2.1 software environment and passed hypothesis tests against other spatio-temporal models. All variables were standardized prior to modeling to facilitate coefficient interpretation. This analysis provides insights into the relative influence of socioeconomic and policy-related factors on the allocation and accessibility of healthcare resources across time and space.

## 3 Results

### 3.1 Spatiotemporal disparities in private and public hospitals

**Figure 3:**
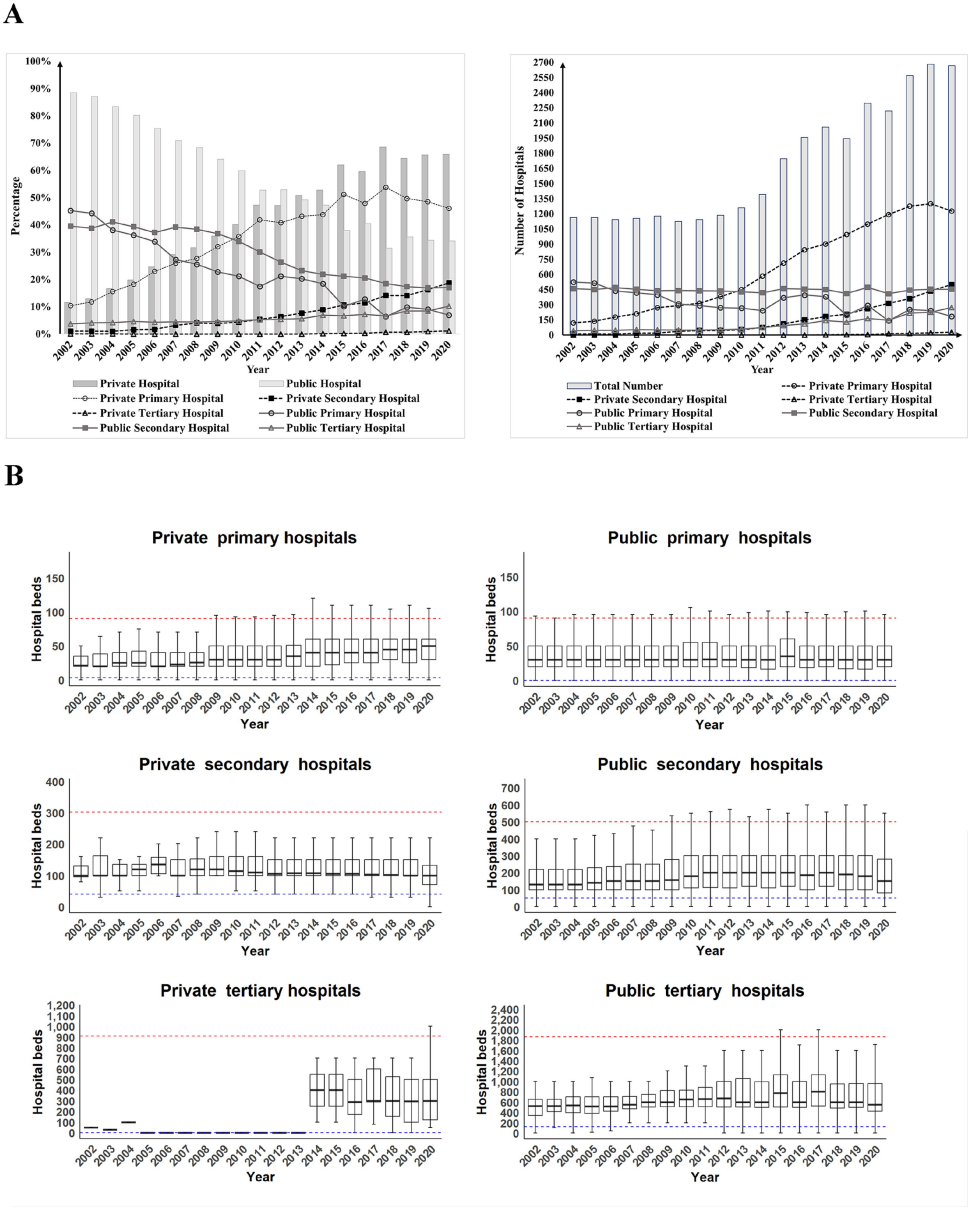
**(A)** Changes in the number and proportional composition of hospitals by ownership type (public vs. private) and hierarchical level (primary, secondary, tertiary) in Sichuan Province from 2002 to 2020. **(B)** Box plots show the distribution of hospital beds by ownership type and hierarchical level over the same period.

Driven by successive policy interventions, medical service coverage in Sichuan province expanded substantially between 2002 and 2020. The total number of private and public hospitals increased from 1,163 to 2,668 (Supplementary Table S1). This growth was predominantly driven by the rapid proliferation of private hospitals, which rose from 134 to 1,757 over the period. The proportion of private hospitals increased by 54.33% (Figure 3A), with private primary, secondary, and tertiary hospitals growing by 35.63%, 17.71%, and 1.00%, respectively. In contrast, public hospitals decreased from 1,029 to 911, reflecting a proportional decline of 54.33%. Reductions were particularly notable in public primary and secondary hospitals, whose numbers declined by 38.3% and 22.53%, respectively. However, public tertiary hospitals, representing top-tier medical institutions in China, grew significantly from 42 to 272, with their proportion increasing by 6.49%.

At the same time, the overall service capacity of the healthcare system improved across different hospital levels (Figure 3B). Among primary hospitals, private hospitals exhibited steady growth in average bed capacity, eventually surpassing public primary hospitals, which remained relatively stable in size. In secondary hospitals, both public and private hospitals experienced an initial increase followed by a decline in average bed capacity, though their peak periods varied. Public secondary hospitals consistently maintained larger average capacities than their private counterparts, which operated close to the minimum threshold of beds required for secondary hospital classification. Private tertiary hospitals remained few in number, with their bed capacity highly variable and largely influenced by individual hospitals. In contrast, public tertiary hospitals demonstrated relatively stable bed capacities, despite long-term fluctuations.

**Figure 4:**
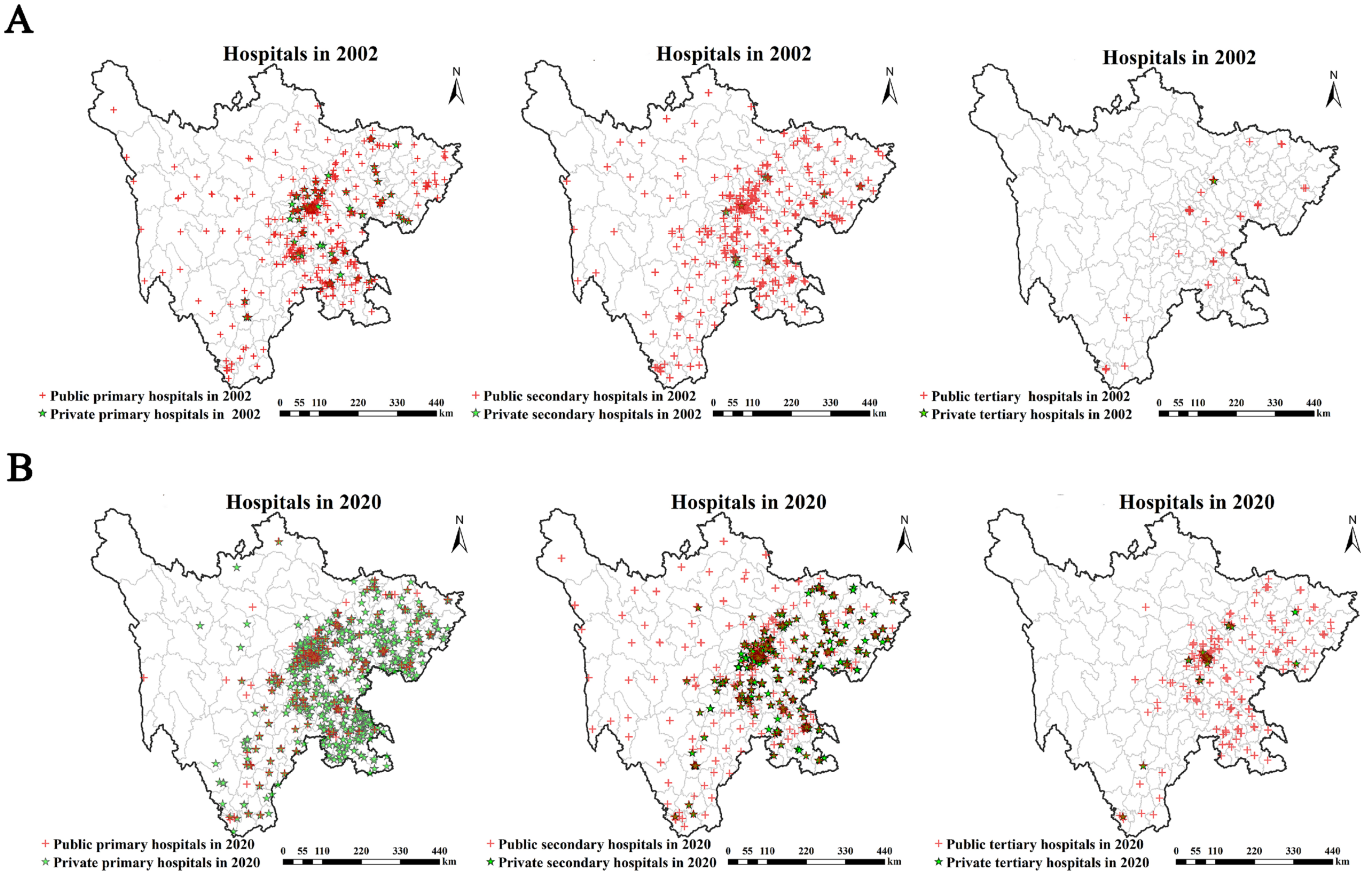
Spatial distribution of public and private hospitals by hierarchical level (primary, secondary, and tertiary) in Sichuan Province, China for the **(A)** 2002 and **(B)** 2020.

Private hospitals in Sichuan exhibited an overall trend of expansion, primarily concentrated in the eastern region of the province (Figure 4). In 2002, private hospitals were relatively scarce, mainly distributed in a few eastern cities like Chengdu and Deyang (Figure 4A). By 2020, their numbers had increased significantly. This growth was particularly pronounced among private primary hospitals, which expanded notably in eastern and southern Sichuan. Private secondary hospitals were predominantly located in eastern cities, competing spatially with public secondary hospitals. The expansion of private tertiary hospitals was the slowest. Although they increased in number by 2020, they remained concentrated in large, developed cities, forming clusters alongside dense aggregations of public tertiary hospitals (Figure 4B). Public hospital growth was especially notable in densely populated areas such as Chengdu and its surrounding cities. While public tertiary hospitals recorded the most substantial increase in absolute numbers, they showed limited spatial diffusion, remaining clustered in the more developed eastern regions. Public secondary hospitals experienced growth in both scale and geographic reach, expanding into underserved districts/counties, particularly in eastern and southern Sichuan. The distribution density of public primary hospitals in western and remote areas declined. This trend can be partially explained by the simultaneous expansion of private hospitals. In 2002, public primary hospitals were more widely distributed, often serving rural and peri-urban transitional zones (Figure 4A). However, by 2020, many of these regions had experienced a shift toward private provision. Spatially, public and private hospitals exhibited clustering in many overlapping regions, highlighting both competitive and complementary dynamics in service delivery (Figure 4B).

**Figure 5:**
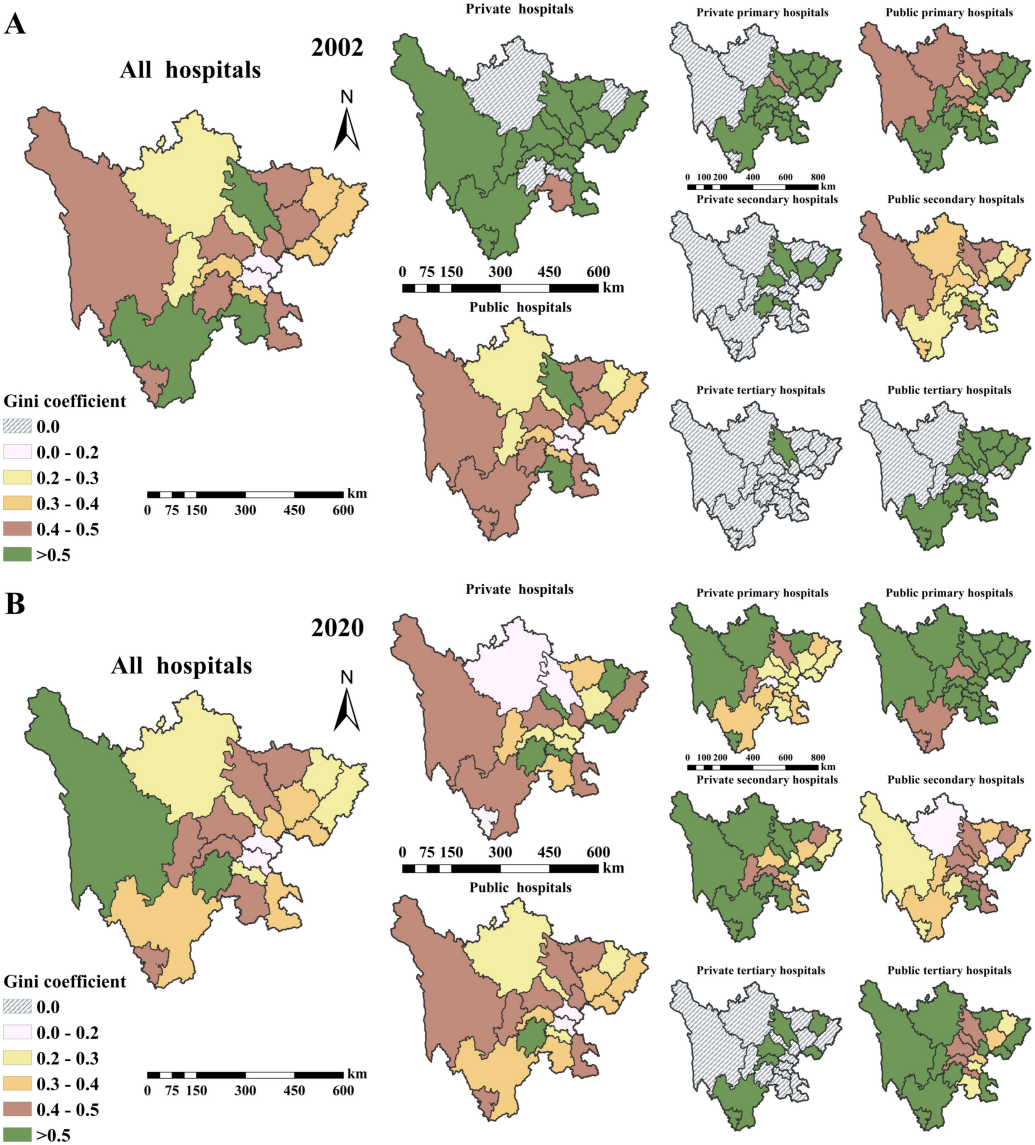
Gini coefficients of spatial accessibility to hospital beds by ownership (public vs. private) and hierarchical level (primary, secondary, and tertiary) in Sichuan Province, China, 2002 **(A)** and 2020 **(B)**. A coefficient of 0 signifies the absence of such hospitals in the prefecture that year.

The spatial distribution of the bed resource Gini coefficient is shown in Figure 5. The study period witnessed a significant improvement in the overall equity of medical bed resources province-wide. This overall progress, however, was accompanied by localized disparities: the spatial equity of public primary hospitals declined in the northeast, and bed resource equity for public secondary hospitals decreased in areas including Chengdu, Leshan, and Luzhou. In contrast, eastern areas saw marked improvements, with the coverage of private primary hospitals, private secondary hospitals and public tertiary hospitals expanding to all prefectures.

### 3.2 Spatial accessibility of hospital beds and its temporal trends

**Figure 6:**
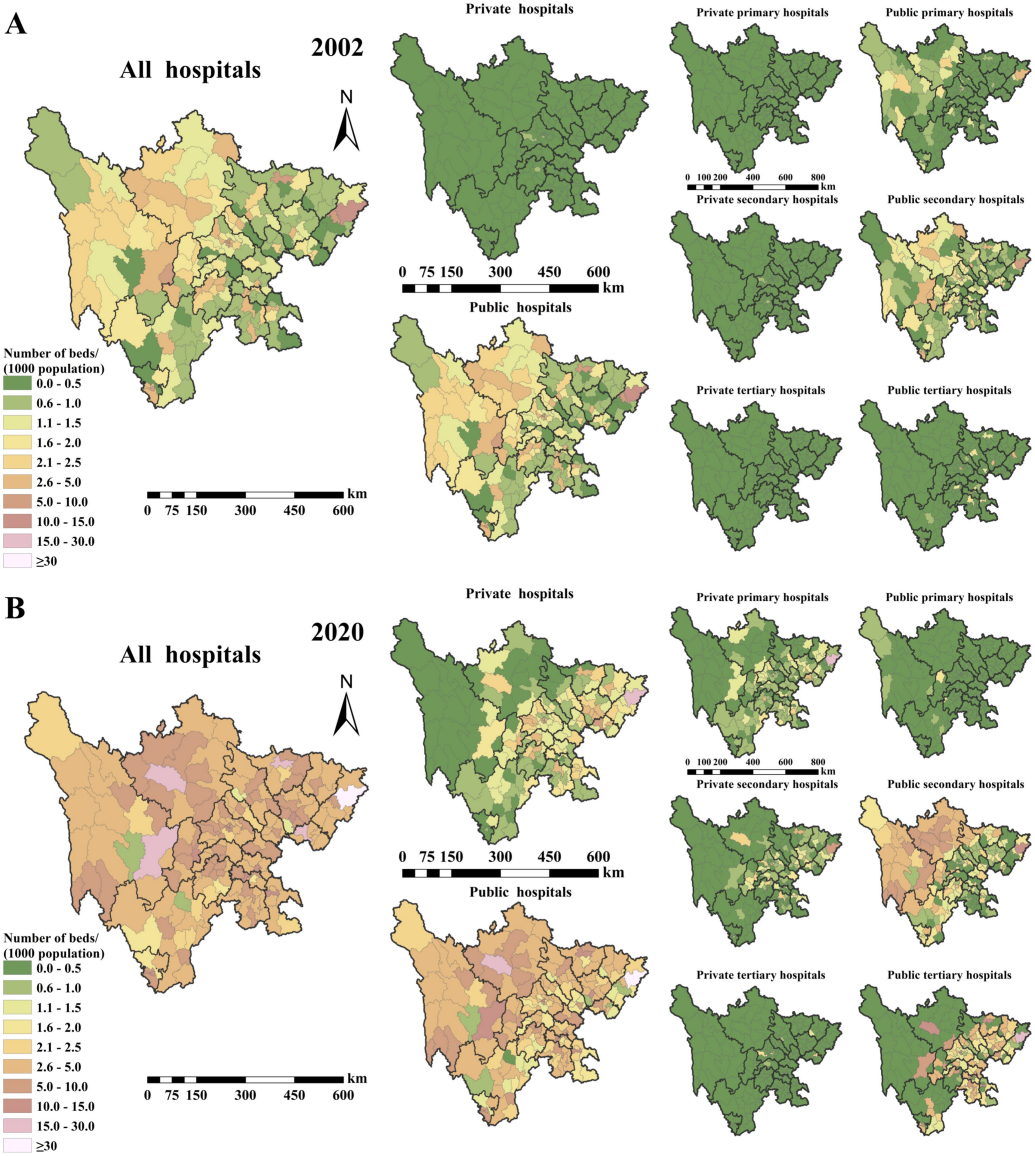
Spatial accessibility of hospital beds by ownership type (public vs. private) and hierarchical level (primary, secondary, and tertiary) in Sichuan Province, China: **(A)** 2002 and **(B)** 2020.

Between 2002 and 2020, the spatial accessibility of hospital beds in Sichuan province improved significantly, reflecting enhanced service capacity across the healthcare system. Figures 6A,B respectively show the spatial accessibility of hospital beds in Sichuan province, China, by ownership type (public vs. private) and hierarchical level (primary, secondary, and tertiary) in 2002 and 2020. In 2002, only 5.46% of districts/counties had more than 4.8 hospital beds per 1,000 population (across all hospital types), whereas by 2020, this figure had risen to 43.72%, indicating substantial progress in overall resource availability. At the beginning of the study period, no private hospital exceeded 1.5 beds per 1,000 population (Figure 6A). However, by 2020, the proportion of districts/counties meeting this threshold for private hospitals increased by 33.33%, with specific growth rates of 6.01% for private primary hospitals, 10.38% for private secondary hospitals, and 1.64% for private tertiary hospitals (Figure 6B). These improvements were largely driven by the expansion of private primary and secondary hospitals. For public hospitals, the proportion of districts/counties with more than 3.3 beds per 1,000 population rose from 12.57% in 2002 to 50.27% in 2020. Disaggregated by hospital level, the proportion of district/county-level public primary hospitals exceeding the 3.3 beds per 1,000 population threshold fell from 0.5% to 0%. In contrast, public secondary and tertiary hospitals experienced notable increases, with the proportion of districts/counties exceeding 3.3 beds per 1,000 population rising from 4.92% to 19.67% and from 1.09% to 20.77%, respectively.

**Figure 7:**
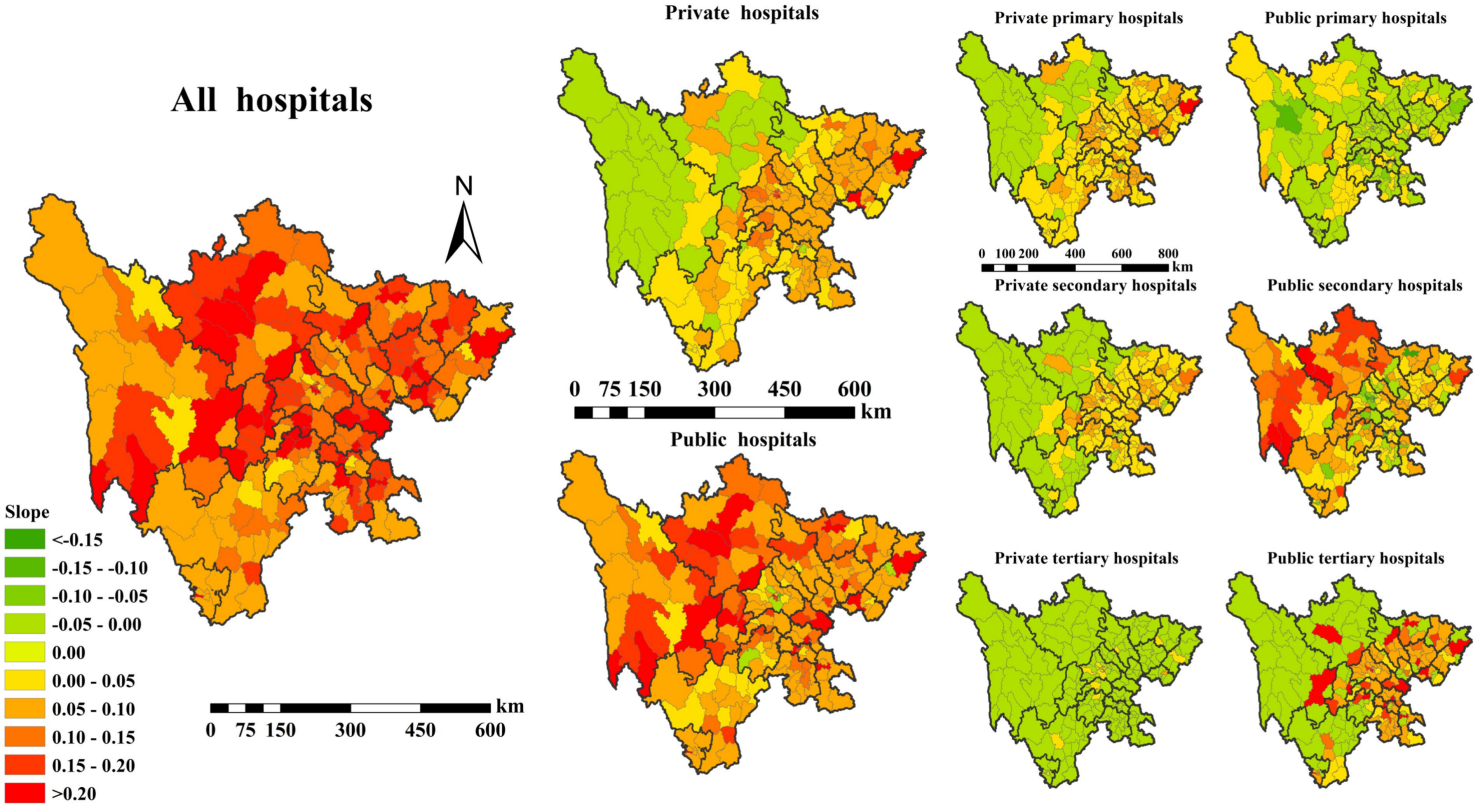
Temporal trends in the spatial accessibility of hospital beds by ownership type (public vs. private) and hierarchical level (primary, secondary, and tertiary) in Sichuan Province, China, from 2002 to 2020.

While healthcare resources, as measured by spatial access to hospital beds, have increased overall, regional disparities persist. Resource availability remains uneven between urban and rural areas, as well as across different geographic regions, with higher concentration in urbanized and economically developed locations. Figure 7 illustrates the temporal trends in the spatial accessibility of hospital beds in Sichuan province, China, by ownership type (public vs. private) and hierarchical level (primary, secondary, and tertiary) from 2002 to 2020. The expansion of private hospitals has been particularly prominent in affluent regions. Spatial accessibility of beds in private primary hospitals increased significantly, with their distribution extending into urban peripheries and economically vibrant zones. Private secondary hospitals showed marked growth in bed numbers in major cities and economic hubs, signaling the private sector’s expanding role in high-end medical services. Public hospital bed capacity remained relatively stable, with moderate increases primarily in urban and densely populated regions. Public primary hospitals exhibited improved spatial accessibility in rural and remote areas, while public secondary and tertiary hospitals continued to maintain higher bed densities in core urban areas. These spatial patterns reflect the influence of economic development, policy interventions, and evolving market demand on the distribution of healthcare resources.

### 3.3 Geospatial interactions between public and private hospitals

**Figure 8:**
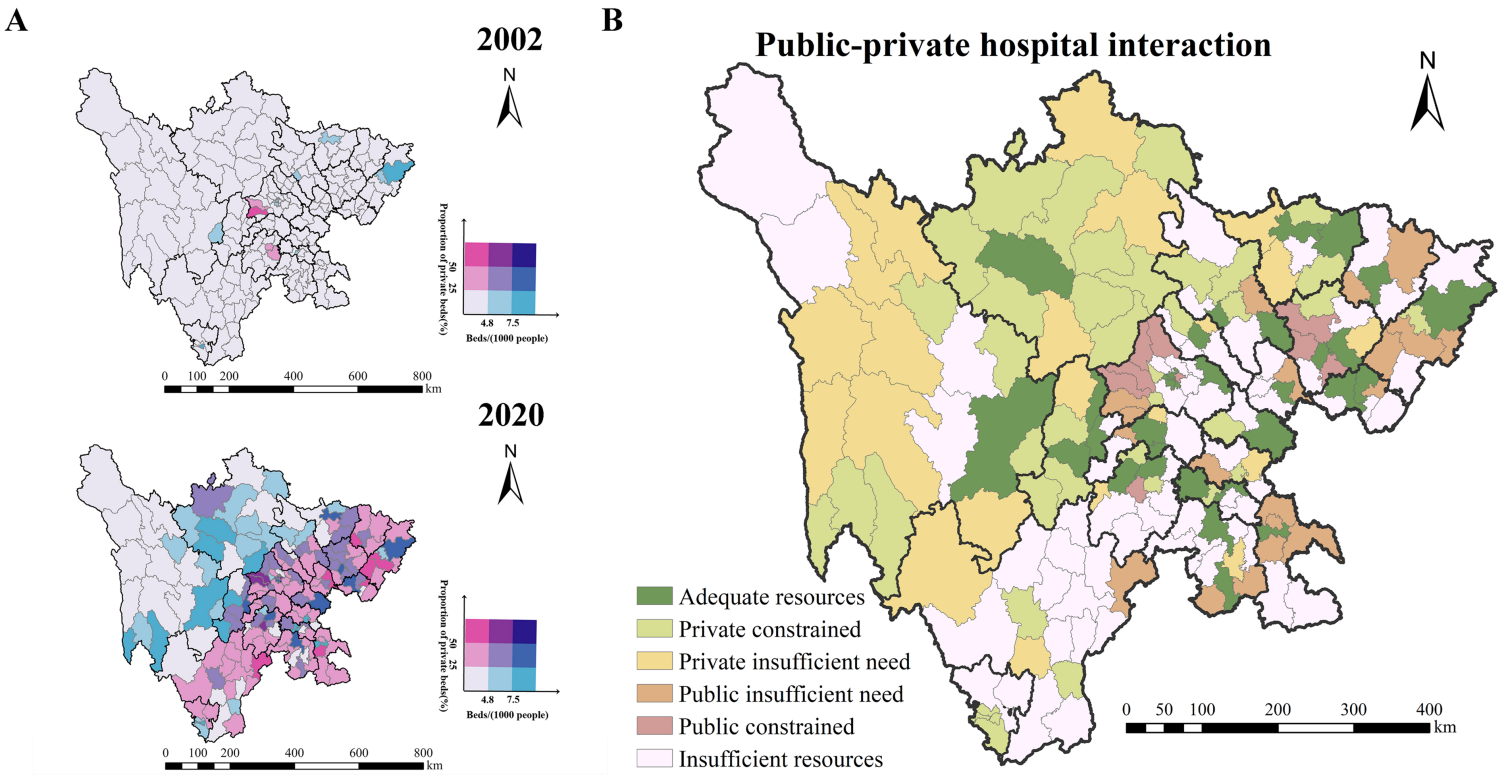
**(A)** Bivariate maps illustrating the relationship between the proportion of private hospitals and spatial accessibility in 2002 and 2020. **(B)** Map depicting geospatial interactions between private and public hospital bed distributions.

In alignment with national China’s policies such as the National Health Service System Plan (2015–2020) and the Guiding Principles for Medical Institution Setup Planning (2021–2025), we constructed a bivariate map (Figure 8A) combining two key indicators: spatial accessibility of hospital beds and the proportion of beds in private hospitals. This analysis aims to identify structural imbalances and regional disparities in the healthcare system under the guidance of these national policy frameworks. In 2002, only 4 districts/counties (2.19%) in Sichuan province had a proportion of private hospital beds reaching 25% or more, while 10 districts/counties (5.46%) had more than 4.8 beds per 1,000 population (a threshold reflecting adequate healthcare service capacity). By 2020, the number of districts/counties with a proportion of private hospital beds ≥ 25% increased to 97 (53.01%). Simultaneously, 80 districts/counties (43.72%) exceeded the 4.8 beds per 1,000 population benchmark, indicating significant expansion in overall medical capacity.

By analyzing the spatial overlap between these two indicators (Figure 8A), distinct regional patterns emerge. In both eastern and western Sichuan, many districts/counties suffer from an overall shortage of hospital beds as well as a low share of private hospital beds, indicating persistent underdevelopment in the healthcare system. Meanwhile, parts of southeastern Sichuan exhibit a high proportion of private hospital beds but still fall below the threshold of 4.8 beds per 1,000 population, reflecting insufficient total capacity despite private sector expansion. These mismatches suggest the need for differentiated resource planning strategies tailored to regional healthcare demands.

The spatial interaction outcomes observed in 2020, shaped by nearly two decades of policy implementation, are illustrated in Figure 8B. Regions with relatively sufficient medical resources tend to be either economically advanced or moderately populated areas, where both public and private hospitals have developed in a complementary manner. Conversely, resource-deficient areas are often found in remote or economically underdeveloped regions, where private hospital development has been constrained and public sector expansion has not compensated adequately. Notably, some urban and economic centers show signs of public hospital space compression, where the growth of private hospitals has partially displaced or limited the expansion of public institutions.

### 3.4 Influences of social determinants on hospital bed allocation

In China, the allocation of medical resources is significantly shaped by key social determinants (47). Specifically, the primary factors influencing the distribution of hospital beds are population density, per capita GDP, urbanization rate, and government health expenditure. These factors generally exhibited positive associations with the number of hospital beds per 1,000 population, aligning with national policies that set clear allocation standards for various tiers of public hospitals. The metric of beds per 1,000 population serves as an important benchmark in guiding resource distribution and reflects broader socioeconomic conditions.

For private hospitals, three specified indicators, population density, per capita GDP and government health expenditure, were strongly and positively associated with bed availability (*p* < 0.001) (Table 1). Beds in private hospitals were predominantly concentrated in densely populated and economically advanced regions. Furthermore, the provider-to-population ratios of private primary and secondary hospitals also demonstrated significant positive correlations with government health expenditure (*p* < 0.001), suggesting fiscal policy incentives may influence private sector expansion.

**Table 1:** Influence of social determinants on hospital bed allocation by ownership type (private vs. public) and hierarchical level (primary, secondary, and tertiary).

Variable	Total	Private				Public			
			Primary	Secondary	Tertiary		Primary	Secondary	Tertiary
Population density (people/km^2^)	0.022***	0.025***	0.007***	0.014***	0.004***	−0.003	−0.004*	−0.013**	0.013***
Per capita GDP (ten thousand)	5.040***	1.608***	0.833***	0.718***	0.057**	3.431***	−0.283*	1.150***	2.570***
Urbanization rate (%)	0.351***	0.076	0.049	0.025	0.000	0.276**	0.057*	0.147	0.072
Basic medical insurance coverage rate (%)	0.003	0.012	0.007	0.005	−0.000	−0.008	−0.011*	0.005	−0.003
Government health expenditure (ten thousand)	0.155***	0.080***	0.042***	0.029***	−0.009***	0.075*	0.013	−0.025	0.087***

*indicates *P* < 0.05, **indicates *P* < 0.01, ***indicates *P* < 0.001.

In contrast, public hospitals exhibited a more complex distributional pattern. Overall, per capita GDP, urbanization rate, and government health expenditure were positively associated with the number of public hospital beds per 1,000 population (*p* < 0.05), suggesting a redistributive policy orientation. A disaggregated analysis by hospital tier revealed heterogeneous trends: public primary hospitals were negatively correlated with per capita GDP (*p* < 0.05), indicating their concentration in economically underdeveloped and sparsely populated regions; public secondary and tertiary hospitals, by contrast, showed positive associations with per capita GDP (*p* < 0.01), reflecting their responsiveness to urban economic development. Moreover, public tertiary hospitals were positively correlated with population density, consistent with their clustering in metropolitan hubs. These findings underscore the differentiated influence of social determinants on the spatial allocation of hospital beds across both public and private sectors, as well as across tiers of service provision.

## 4 Discussion

Achieving UHC in low- and middle-income countries is hindered by the uncoordinated development between public and private hospitals. This study focuses on Sichuan Province, China, to examine the co-evolution of public and private hospitals from 2002 to 2020, under the influence of long-term policy guidance. We analyze the spatiotemporal distribution disparities, geospatial interactions, and social determinants that shape their development, with the aim of optimizing resource allocation for UHC. Our contribution lies in providing empirical evidence of the structural imbalances in hospital resource allocation and the socio-policy factors that hinder UHC progress, particularly in underserved regions. Next, we explore the specific findings and policy implications of our study.

### 4.1 Policy-driven evolution of the healthcare resource landscape

The allocation of medical resources is undergoing a profound structural transformation, guided by national and regional health policies. This process has led to significant heterogeneity between public and private hospitals, particularly in terms of hospital grade and spatial distribution. Since the implementation of China’s new healthcare reform in 2009, private hospitals at lower tiers have experienced considerable growth in both number and service coverage. However, their expansion remains concentrated in economically developed regions, such as eastern and southern Sichuan. In contrast, the growth of private tertiary hospitals has been slow and primarily confined to eastern Sichuan, highlighting persistent regional and hierarchical challenges in the development of high-level private healthcare providers.

Public hospitals have followed a pattern of hierarchical upgrading, characterized by a stepwise progressive elevation in service levels. As a result, the number and proportion of public primary and secondary hospitals have steadily declined, while public tertiary hospitals have gradually become dominant, geographically concentrated in urban cores. This trend aligns with policies that strictly control the scale of public hospitals while reserving space for the development of private hospitals. From the perspective of optimizing the hierarchical healthcare system, the rise of private primary hospitals in eastern Sichuan has, to some extent, alleviated the decline in grassroots service capacity resulting from the upgrading of public hospitals. However, in remote districts/counties and western Sichuan, medical resources still heavily depend on public primary and secondary hospitals, exacerbating regional disparities in access to healthcare services (48).

### 4.2 Structural imbalances in the development of private hospitals

Although private hospitals in China have achieved considerable expansion in total bed capacity, this growth has been accompanied by significant structural imbalances. In Sichuan province, private hospitals accounted for 53.01% of total beds in 2020, significantly surpassing the national average of 28.62%. This rapid increase demonstrates the effectiveness of policy in stimulating private sector participation. However, the number of private hospital beds per 1,000 population in Sichuan province remains low at just 1.38, falling short of the policy target of 1.5, with only 33.33% of districts/counties meeting the goal. In contrast, public hospitals reached 4.12 beds per 1,000 population, surpassing the target. This disparity is rooted in differences in hospital grade structure: 98.35% of private hospital beds are provided by primary and secondary hospitals, with few high-level (tertiary) hospitals. In comparison, approximately 69.65% of public hospital beds are located in tertiary hospitals. This pattern holds at the national level, with private hospitals providing only 1.18 beds per 1,000 population compared to 5.0 in public hospitals.

This imbalance arises from policy misalignments and short-term market tendencies. Current policies emphasize quantitative targets such as the proportion of private hospital beds and beds per 1,000 population, but fail to provide clear guidance on hospital grade structure and service tier development. As a result, local governments have permitted disorderly expansion of low-end private hospitals, neglecting regional equity and service quality. This has led to a pattern of scale expansion without corresponding improvements in quality. Moreover, private hospitals, driven by short-term profits, frequently concentrate on low-risk, high-profit specialties, such as obstetrics and cosmetic medicine, rather than addressing fundamental healthcare gaps in underdeveloped areas like Western Sichuan. Consequently, private hospitals fail to fully fulfill their role in supplementing public healthcare.

International comparisons further illustrate the structural contradictions. In South Korea, where the healthcare system is predominantly privatized, private hospitals provided 11.43 beds per 1,000 population in 2020. However, market-driven competition often leads to the concentration of resources in profitable specialties, while primary care is underprovided, a situation similar to China’s pattern of low-end concentration and high-end shortage. In contrast, Japan’s well-established tiered diagnosis and treatment system enables rational distribution of private hospitals across different service levels. This balance allows private hospitals to supplement primary care while preventing excessive resource concentration in high-profit sectors, maintaining 9.15 private hospital beds per 1,000 population and optimizing the public-private resource (49).

Currently, China’s private hospitals face functional ambiguity, with an excess of low-end infrastructure and insufficient high-end service capacity, resulting in overall inefficiency. Institutional reform is urgently needed to clarify the functional roles of private hospitals: steering certain facilities toward specialized, high-complexity care while directing others to strengthen community-based primary care. Structural planning must be enhanced by integrating hospital tier and service capability into evaluation frameworks and by fostering synergy between private and public providers within a tiered healthcare service system. Evidence-based analytical tools should be embedded into health planning and decision-making processes to guide the strategic resource allocation (50–52). Collectively, these reforms would help establish a well-differentiated, multi-level healthcare system capable of optimizing resource distribution and improving overall system efficiency.

### 4.3 Differentiated impact of social factors and achievement of UHC goals

The allocation of hospital beds in both private and public hospitals reveals distinct patterns shaped by underlying social and economic determinants (53). The number of beds in private hospitals is positively correlated with population density and per capita GDP, reflecting the inherently profit-driven nature of the private sector. This results in the natural flow of resources to areas with stronger economic attractiveness in the absence of targeted spatial planning policies. On the other hand, public hospitals are more strongly influenced by policy guidance. In economically developed cities, government investment tends to focus on upgrading tertiary hospitals, which further concentrates them in urban cores, reinforcing a Matthew effect cycle where high-quality resources attract more patients (46, 54). As a result, developed urban areas like Chengdu not only benefit from high-quality public hospital resources but also have access to more personalized care from private healthcare providers.

In contrast, remote and less developed regions in western Sichuan still rely on primary-level public hospitals to meet basic healthcare needs. However, these institutions often face significant challenges in terms of service quality and resource capacity, leading to persistent disparities in healthcare accessibility and effectiveness across regions. This is particularly problematic for vulnerable groups in low-GDP areas, such as Garzê, Ngawa, and Liangshan, where residents face higher disease burdens and lower health literacy, yet struggle to access advanced medical services. With private hospitals failing to effectively supplement primary care, these regions remain almost entirely dependent on the public system, posing serious challenges to achieving UHC.

Although government health expenditures (positively associated with the provider-to-population ratio in private primary and secondary hospitals) have incentivized the growth of low-level private hospitals, these financial stimuli have not addressed the underlying structural imbalances. Therefore, policy interventions must shift toward more targeted, pro-poor resource allocation (55). In regions similar to western Sichuan, direct government investment is necessary to upgrade public hospital infrastructure, accompanied by incentives to attract private institutions to establish non-profit or subsidized services.

### 4.4 Policy recommendations for LMICs hospital development

The evolution of Sichuan’s healthcare system reflects a public-private bifurcation and regional imbalance, which requires differentiated policy interventions and strategic resource reallocation. Based on the current situation in LMICs, the following region-specific policy recommendations are proposed:

In resource-scarce regions with low private hospital coverage, governments should reduce barriers to private medical practice through fiscal subsidies, land-use incentives, and tax reductions. While guiding social capital toward establishing primary-level private hospitals, public hospitals should focus on providing essential medical services. This approach can prevent a vicious cycle wherein public hospitals undergo continuous upgrades while underserved areas experience a persistent absence of private medical services (56). Additionally, the WHO Digital Health Toolkit can be utilized to attract social capital through public-private partnerships (PPPs) to build digital health infrastructure at the primary level. For example, in remote areas of Africa, collaborative efforts can establish mobile health data platforms that connect private clinics to public health surveillance systems. This not only lowers the government’s sole construction costs but also enhances the responsiveness of grassroots medical services.

In regions with limited resources but substantial private hospital coverage, such as parts of eastern Sichuan, policy should focus on regulating and supporting private hospitals, the government should guide private hospitals to fill the resource gaps or functional shortcomings of public hospitals, leveraging market mechanisms to optimize resource allocation. By promoting public-private collaboration and resource sharing, it is possible to resolve the contradiction of weak technical capacity in private hospitals and resource strain in public hospitals, ultimately helping to develop mid- to high-level private hospitals and improve residents’ access to quality healthcare.

In areas like sub-Saharan Africa where resources are inadequate and private involvement is minimal, governments must strengthen infrastructure by building new public hospitals, while also using fiscal incentives to stimulate private market engagement. In parallel, efforts to cultivate and attract medical talent are essential to support service provision and meet basic health demands (57).

In regions such as Rio de Janeiro, Brazil, where private medical resources are abundant but public hospitals lag behind, governments should prioritize funding to help public hospitals improve infrastructure, acquire medical equipment, and recruit skilled professionals (58). By establishing smart health platforms, public hospitals can share advanced equipment with private hospitals, while private hospitals can contribute public health resources to the public system. At the same time, digital monitoring tools can be used to track the utilization of regional medical resources in real time, facilitating public-private resource integration and complementary service development (33, 59).

### 4.5 Limitations

This study has several limitations. First, due to data constraints, our analysis relied solely on structural metrics, such as the number of hospitals and bed capacity, while missing key service quality indicators like mortality and bed utilization rates, which could provide a more comprehensive assessment of healthcare effectiveness. Additionally, important indicators reflecting the effectiveness of the tiered healthcare system, such as readmission rates, were unavailable. Second, despite cross-verification with official sources, early hospital data may still be underreported or not reflect real-time updates in hospital grading. Third, the use of district/county-level units may introduce aggregation biases, such as the modifiable areal unit problem (MAUP), potentially masking internal disparities in resource distribution. Finally, the spatial method (potential path area, PPA) used does not consider dynamic factors like travel time or transportation modes, which could affect the accuracy of urban–rural accessibility differences. Future research should incorporate more granular data, dynamic accessibility models, and service quality metrics to overcome these limitations.

## 5 Conclusion

This study combines spatiotemporal analysis with a social determinants perspective to examine the co-evolution of public and private hospitals at different levels under long-term policy guidance, uncovering structural contradictions and regional imbalances in their coordinated development. Empirical evidence from Sichuan, China, shows that while private hospitals have expanded bed capacity, they face significant hierarchical issues, namely a shortage of high-end services alongside a surplus of low-end resources, with a spatial concentration in economically developed regions. In contrast, public hospitals have strengthened tertiary hospitals through a stepwise upgrading process, but basic medical supply remains insufficient in remote regions. This dualistic structure has resulted in disparities in both the quantity and quality of healthcare resources, posing major challenges to achieving UHC. To address similar challenges in LMICs, this study proposes a spatial equity-oriented approach to optimize medical resource allocation. First, establish a targeted tiered policy framework to promote functional complementarity between public and private hospitals through differentiated measures. Second, include hospital grade and service capacity in policy evaluation metrics, moving beyond a focus on bed numbers in expansion strategies. Third, develop collaborative models between public and private hospitals that are tailored to regional needs, especially in underserved areas. China’s experience highlights that integrating social determinants into policy design and establishing a multi-tiered medical service system is essential for the optimal allocation of medical resources and the sustainable achievement of UHC.

## Data Availability

The raw data supporting the conclusions of this article will be made available by the authors, without undue reservation.
