# Supplementary material for: Co-evolution of private and public hospitals: spatiotemporal disparities, geospatial interactions, and social determinants over 19 years in Sichuan, China
Source: Front Public Health. 2025 Dec 8;13:1644657. doi: 10.3389/fpubh.2025.1644657 (PMC12722804; doi:10.3389/fpubh.2025.1644657)
Supplement: Supplementary file 1 [file Data_Sheet_1.pdf]

Supplementary Material

1 Supplementary Tables

Supplement Table S1 The number of hospitals in Sichuan Province from 2002 to 2020 categorized by ownership type (public and private) and hierarchical level (primary, secondary, tertiary)

Year	Total	Private				Public			
			Primary	Secondary	Tertiary		Primary	Secondary	Tertiary
2002	1,163	134	121	12	1	1,029	526	460	43
2003	1,164	150	137	12	1	1,014	515	451	48
2004	1,144	190	178	11	1	954	436	470	48
2005	1,155	229	211	18	0	926	419	454	53

2006	1,178	290	270	20	0	888	399	438	51
2007	1,126	328	292	36	0	798	307	441	50
2008	1,143	362	316	46	0	781	292	439	50
2009	1,187	426	380	46	0	761	270	437	54
2010	1,260	505	451	54	0	755	267	428	60
2011	1,393	658	583	75	0	735	243	419	73
2012	1,747	823	712	111	0	924	370	460	94
2013	1,957	995	845	150	0	962	397	455	110
2014	2,061	1,087	902	183	2	974	380	451	143
2015	1,945	1,206	995	207	4	739	197	412	130
2016	2,297	1,367	1,099	262	6	930	293	472	165
2017	2,219	1,520	1,193	313	14	699	142	410	147

2018	2,569	1,654	1,276	361	17	915	252	446	217
2019	2,682	1,760	1,301	436	23	922	242	454	226
2020	2,668	1,757	1,228	500	29	911	185	454	272

Supplement Table S2 LM test and robustness test results of the spatiotemporal lag model and spatiotemporal error model

Hospital types	STLM		STEM	
	LM-Lag	LM-Error	LM-Lag	LM-Error
Total	0.326	0.987	0.326	0.497
Private hospital	0.941	0.998	0.941	0.954
Private primary hospital	0.402	0.995	0.402	0.488
Private secondary hospital	0.636	0.999	0.636	0.701
Private tertiary hospital	0.402	0.995	0.402	0.488
Public hospital	0.803	0.999	0.803	0.873
Public primary hospital	0.561	0.962	0.561	0.986
Public secondary hospital	0.738	0.780	0.738	0.809
Public tertiary hospital	0.910	0.967	0.910	0.940

Note: The *P*-values for the LM tests of both STLM and STEM are greater than 0.05

Supplement Table S3 Test results for multicollinearity of explanatory variables

Variable	VIF
Population density (people/km²)	1.502
Per capita GDP (ten thousand)	2.813
Urbanization rate (%)	2.634
Basic medical insurance coverage rate (%)	2.118
Government health expenditure (ten thousand)	1.737
